# Global Gene Transcriptome Analysis in Vaccinated Cattle Revealed a Dominant Role of IL-22 for Protection against Bovine Tuberculosis

**DOI:** 10.1371/journal.ppat.1003077

**Published:** 2012-12-27

**Authors:** Sabin Bhuju, Elihu Aranday-Cortes, Bernardo Villarreal-Ramos, Zhou Xing, Mahavir Singh, H. Martin Vordermeier

**Affiliations:** 1 Helmholtz Centre for Infection Research, Braunschweig, Germany; 2 Lionex Diagnostics Ltd, Braunschweig, Germany; 3 Department of Bovine Tuberculosis, Animal Health and Veterinary Laboratories Agency, Weybridge, Addlestone, Surrey, United Kingdom; 4 McMaster University, Hamilton, Ontario, Canada; McGill University, Canada

## Abstract

Bovine tuberculosis (bTB) is a chronic disease of cattle caused by *Mycobacterium bovis*, a member of the *Mycobacterium tuberculosis* complex group of bacteria. Vaccination of cattle might offer a long-term solution for controlling the disease and priority has been given to the development of a cattle vaccine against bTB. Identification of biomarkers in tuberculosis research remains elusive and the goal is to identify host correlates of protection. We hypothesized that by studying global gene expression we could identify *in vitro* predictors of protection that could help to facilitate vaccine development. Calves were vaccinated with BCG or with a heterologous BCG prime adenovirally vectored subunit boosting protocol. Protective efficacy was determined after *M. bovis* challenge. RNA was prepared from PPD-stimulated PBMC prepared from vaccinated-protected, vaccinated-unprotected and unvaccinated control cattle prior to *M. bovis* challenge and global gene expression determined by RNA-seq. 668 genes were differentially expressed in vaccinated-protected cattle compared with vaccinated-unprotected and unvaccinated control cattle. Cytokine-cytokine receptor interaction was the most significant pathway related to this dataset with IL-22 expression identified as the dominant surrogate of protection besides INF-γ. Finally, the expression of these candidate genes identified by RNA-seq was evaluated by RT-qPCR in an independent set of PBMC samples from BCG vaccinated and unvaccinated calves. This experiment confirmed the importance of IL-22 as predictor of vaccine efficacy.

## Author Summary

Bovine tuberculosis (bTB) is a chronic disease of cattle caused by *Mycobacterium bovis*, a member of a complex group of pathogens that also includes the bacterium causing human tuberculosis, *M. tuberculosis*. Vaccination of cattle might offer a long-term solution for controlling the disease and priority has been given to the development of a cattle vaccine against bTB. In addition to the relevance for the veterinary field, cattle can also serve as useful pre-clinical model for the development of human vaccines. Identification of biomarkers in tuberculosis research remains elusive and the goal of this study was to identify host markers that allow the prediction of vaccine success. In this experiment, the outcome of vaccination was measured against relevant endpoints based on disease severity. Our results, generated using the state-of-the-art methodology of transcriptome sequencing defined a dominant role of the cytokine IL-22 for predicting vaccine success. We therefore identified a predictive biomarker that can be used in future experiments and also highlighted the role of T cells producing this cytokine in protective immunity.

## Introduction

Bovine tuberculosis (bTB) is a chronic disease of cattle caused by *Mycobacterium bovis*, a member of the *Mycobacterium tuberculosis* Complex group of bacteria. bTB is a significant economic burden to the agricultural industries worldwide. It has been estimated that more than 50 million cattle are infected worldwide with *M. bovis* resulting in economic losses of approximately $3 billion annually [1]. The incidence of bTB in Great Britain (GB) has been on the increase since 1988 [2], [3] and by 2010 approximately 10.8% of the national herd were under TB restriction. Thus, in the last 10 year £500 million have been spent to control bTB in England and it is estimated that up to £1 billion will have to be spent over the next decade in the absence of alternative control strategies. Consequently, the bTB control and eradication programs based on test and slaughter policies will unlikely be sufficient without further control measures. BCG vaccination of cattle induced protective immunity to experimental bTB by reducing primarily the degree of pathology, yet failed to induce more than 50% protection in the majority of field experiments against natural infection [4]. Moreover, in cattle BCG vaccination can induce protective immunity in some calves that results in the resolution of *M. bovis* infection [5], a finding also mirrored in humans infected with *M. tuberculosis* [6], [7]. The most promising vaccination strategies identified to date have mostly involved improving upon BCG vaccination rather than replacing it. BCG therefore remains the prototype vaccine against which to judge the efficacy of any novel vaccine strategies. Several strategies have been implemented to improve the efficacy of BCG, namely the use of subunit vaccines in the form of DNA vaccines, protein subunit vaccines administered with a suitable adjuvant, live recombinant vaccines such as attenuated recombinant viruses expressing mycobacterial antigens, or over-expressing genes in BCG that are deleted or are under-expressed in BCG [8].

At present there are no reliable immunological correlates of protection (i.e. predicting the success of vaccination after completion of the vaccination protocol but before exposure to the pathogen) for TB. The identification of such predictive biomarkers would greatly facilitate the development of a vaccine against bTB. Previous data from our group [5] and others [9], [10] have demonstrated that BCG vaccination of cattle induced an immunological profile characterised by cell mediated immunity with predominant and strong INF-γ production.

The successful development of a new vaccine against TB would be greatly supported by reliable and well-defined predictors and correlates of protection that would be use as go/no-go decision gateway points for further candidate vaccine testing. In order to identify biomarker patterns, it is essential to use tools that can measure an integrated host response rather than isolated characteristics of the adaptive immune response. The tools for quantifying the changes in gene expression have been changing with time and technological development, from limited capabilities and time consuming methods (for example Northern blotting, RT-PCR, SAGE) to effective, fast and high-throughput whole transcriptome quantification using microarrays and new generation sequencing methods (NGS) [11].

## Results

### Characterisation of calves selected for RNA-seq analysis

**Figure 1:**
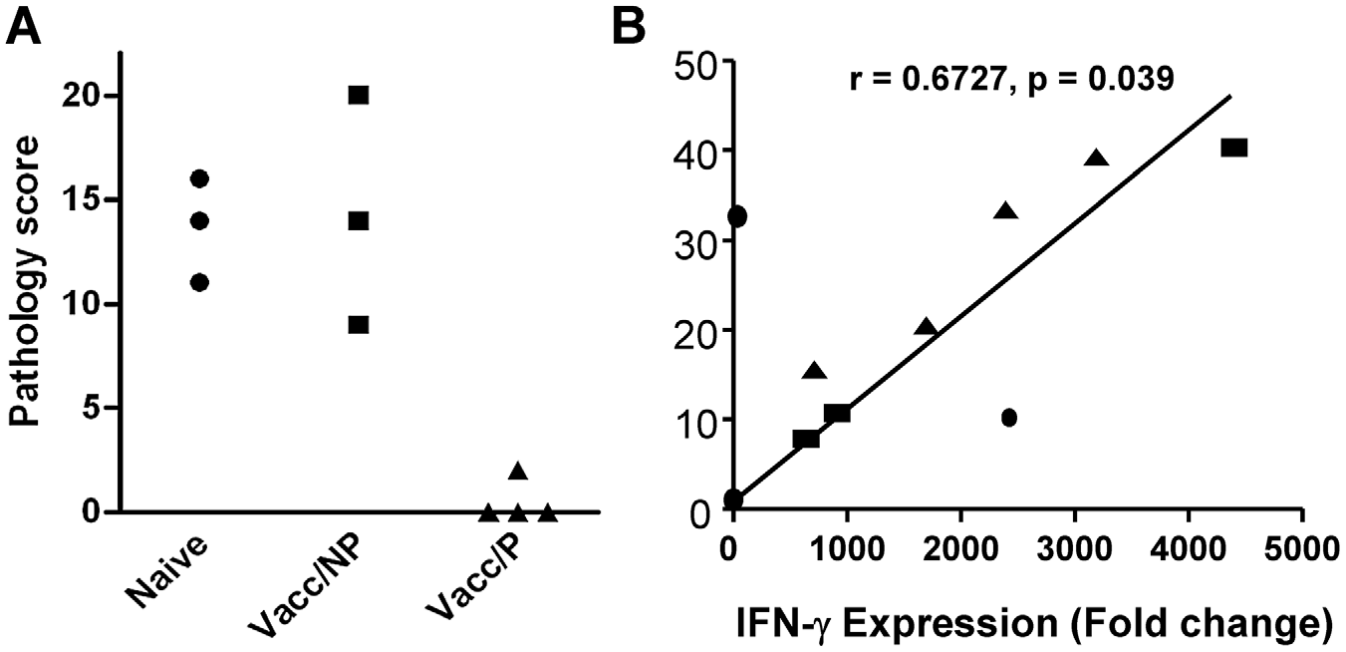
Protection and *in vitro* IFN-γ responses prior to challenge. A. Individual pathology scores are shown for the animals used in this study. Naïve animals = no vaccination, Vacc/NP = vaccinated calves that were not protected; Vacc/P = vaccinated calves that were protected. B. Correlation of IFN-γ protein production in culture supernatants measured by Bovigam ELISA (y-axis) and *ifn-γ* gene expression as determined by deep sequencing (x-axis). Data are shown from PPD-B stimulated PBMC from individual animals. Supernatants and RNA were prepared after 24 h culture.

In the first experiment, groups of 5 calves each were vaccinated with BCG or with a heterologous prime-boost protocol of BCG priming followed by Ad85 boosting. As expected, both BCG and BCG/Ad5 vaccination resulted in significant protection against *M. bovis* challenge compared to uninfected animals. Although a trend towards improved protection using BCG/Ad5 was observed, this difference was not statistically significant (Mean pathology scores [± SEM] for naïve cattle, BCG and BCG/Ad85 vaccinated, respectively: 12.3 [1.8], 7.2 [3.5], 5.6 [2.6]). As in previous experiments, we also observed in each vaccine group animals that were protected, i.e. presented without or with only minor visible pathology at post-mortem, whilst other vaccinated animals showed pathology undistinguishable from unvaccinated control animals. Therefore we selected 4 animals across the vaccination groups that were protected (2 BCG and 2 BCG/Ad85 vaccinated calves), 3 animals that were not protected (2 BCG and 1 BCG/Ad85A vaccinated calves) and 3 animals from the unvaccinated control group for the RNA sequencing experiment. The pathology scores of the selected animals are shown in Figure 1A.

Heparinised blood from these animals was collected 14 weeks post-BCG priming before *M. tuberculosis* challenge. PBMC were prepared and stimulated with PPD-B. After 24 h culture, supernatants were collected for IFN-γ ELISA and RNA prepared from cell pellets for transcriptome analysis by RNA-seq. As in previous experiments, *in vitro* IFN-γ production correlated well with its transcription level [12], [13] (Figure 1B). However, the IFN-γ responses of individual animals did not correlate with their protection status again highlighting the need for additional predictive markers of vaccine efficacy to complement IFN-γ (data not shown).

### Definition of biomarkers of protection

**Figure 2:**
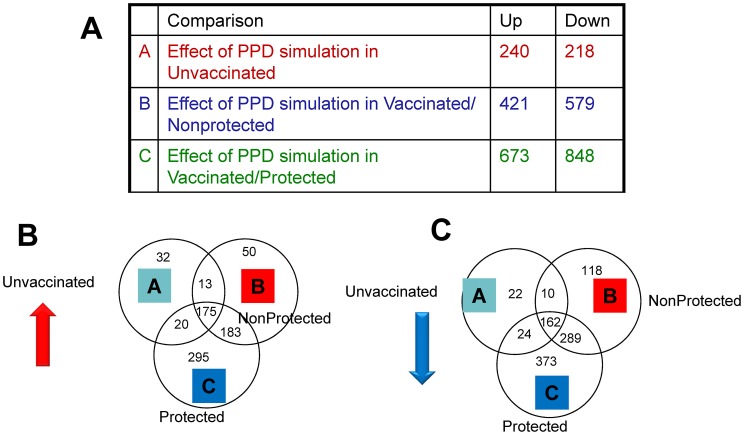
Results of RNA-Seq analysis. A. Signficantly modulated genes in the three treatment groups. B. Venn diagrams of genes significantly up-regulated and (C) down-regulated genes after vaccination but prior to *M. bovis* challenge. A. Fold change compared to unstimulated PBMC (medium controls) of PPD-B stimulated PBMC compared to medium controls from unvaccinated, naïve calves (group 1), vaccinated/non-protected (group 2), and vaccinated/protected calves (group 3).

Firstly, we prepared gene lists of those genes that were significantly modulated (>2-fold change, p<0.05) after PPD-B stimulation of PBMC from the three groups of animals. In total 240, 421 and 673 genes were up-regulated significantly in PBMC from unvaccinated, vaccinated/un-protected, and vaccinated/protected, respectively (Figure 2A). Interestingly, 295 of these genes were significantly up-regulated exclusively in PBMC isolated from the vaccinated/protected calves (Figure 2B). Amongst the genes most strongly up-regulated in the group of protected animals were those encoding IL-22, IFN-γ, CCL3, IL-13, MT3, (see table S1 for a full list of these genes).

In contrast, 218, 579 and 848 genes, respectively were down-regulated after PPD-B stimulation of PBMC isolated from these three groups (Figure 2A), with 373 of these genes exclusively and significantly down-regulated in vaccinated and protected calves (Figure 2B). Amongst the genes most down-regulated in vaccinated/protected calves were those encoding VCAM1, CXCL12 and CCL14. A list of these 373 genes is provided in Table S1.

**Figure 3:**
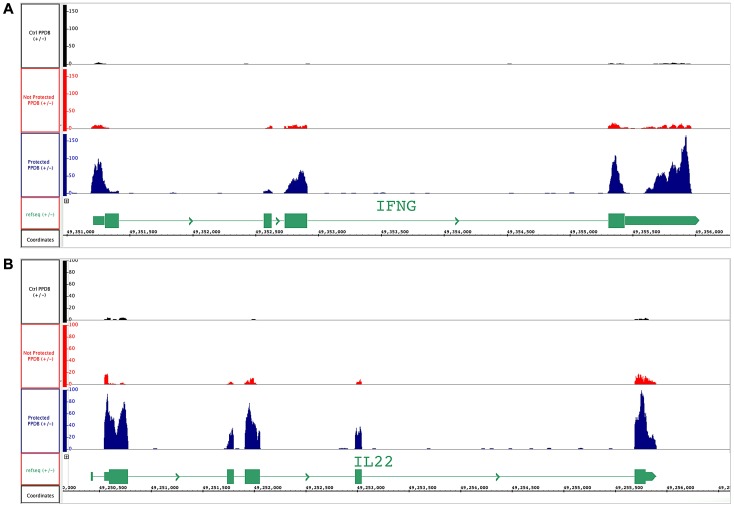
BCG-vaccinated and control cattle samples mapped to *ifn-γ* and *il-22* genes. Visualization by IGB of RNA sequencing reads of representative PPD-B stimulated PBMC from vaccinated-protected, vaccinate-un-protected and non-vaccinated control cattle. Y-axis shows the number of reads covering each base along the transcript in RPKM expression values for each sample. Black track: unvaccinated control cattle; red track: vaccinated/un-protected cattle and blue track: vaccinated/protected cattle. The schematic representation of transcript for (A) *ifn-γ* and (B) *il-22* is show in green at the bottom of the each figure; the boxes show the exons of the gene.

Figure 3 shows visualization of the expression of IL-22 and IFN-γ using Integrated Genome Browser (IGB, [14]) for one representative PPD-B stimulated PBMC sample each from vaccinated/protected, vaccinated/not-protected and control calves. After sequencing, each of the reads was mapped against the reference genome (*Bos taurus*) and the chromosomal positions of the two genes are shown. The histograms represent the reads mapped in their exact positions for *ifn-γ* (Figure 3A) and *il-22* (Figure 3B).

### Functional analysis

**Figure 4:**
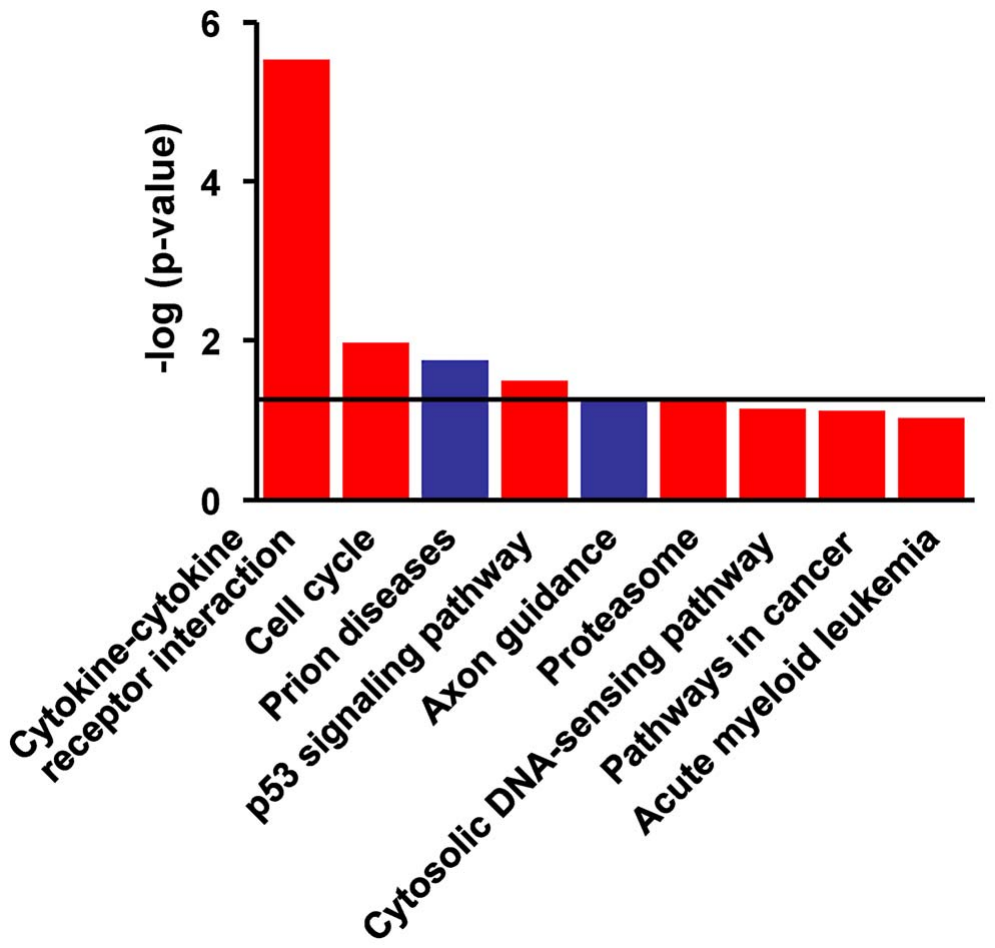
Functional networks most significanty modulated in PPD-B stimulated PBMC from vaccinated/protected calves. Visualisation of the trend and significance of each network: Red bars = up-regulation; blue bars = down-regulation of network. Horizontal bar: p = 0.05.

**Figure 5:**
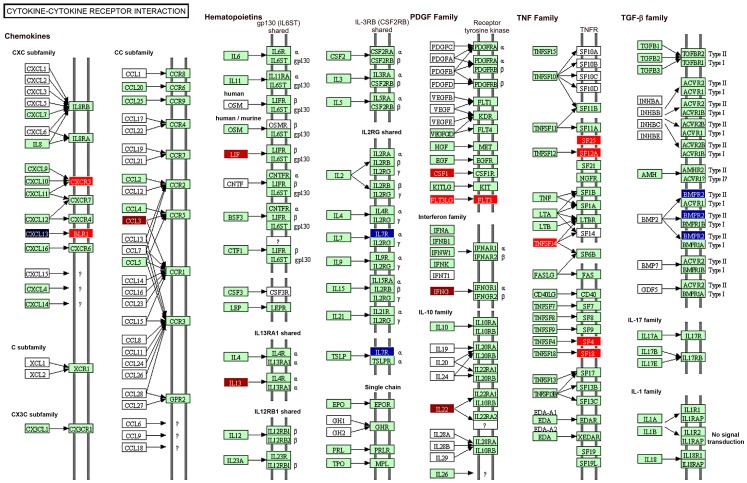
Schematic representation of the genes involved in the cytokine-cytokine receptor interaction. Colour codes indicate genes that were significantly modulated in vaccinated/protected calves prior to *M. bovis* challenge. Red colour: up-regulated genes; blue colour: down-regulated genes. Darkness of colour indicates level of gene modification.

Functional analysis using the DAVID Bioinformatics Database was performed on the 668 genes that were significant regulated in PBMC from vaccinated and protected calves. Of the 295 genes that were significantly up-regulated the pathways most related with this data set were the Cytokine-Cytokine Receptor Interaction pathway (P = 2.89×10^−6^), Cell Cycle, p53 Signalling, and Proteasome-Associated Pathways (all P<0.05) (Figure 4). On the other hand, Prion Disease and Axon Guidance Pathways were populated with down-regulated genes (P<0.05, Figure 4). Associations that did not reach statistical significance were also demonstrated with the Cytosolic DNA Sensing, Cancer and Acute Myeloid Leukemia pathways (Figure 4). Figure 5 depicts the genes and gene product interactions within the -Cytokine Receptor Interaction pathway that were most significantly related to protection. The genes encoding IL-22, IFN-γ, CCL3 and IL13 were most strongly up-regulated (see also Table S1), again highlighting the dominant role of IL-22 in protection.

### Validation of the *il-22* association with protection

To validate the results obtained from RNA-seq, we obtained RNA samples from PBMC collected from an independent experiment. This experiment (referred to as experiment 2 in the methods section) comprised animals neonatally vaccinated with BCG vaccinated that were 7 months later challenged with *M. bovis* (n = 16) and an age-matched control group of naive calves (n = 8) infected at the same time.

**Figure 6:**
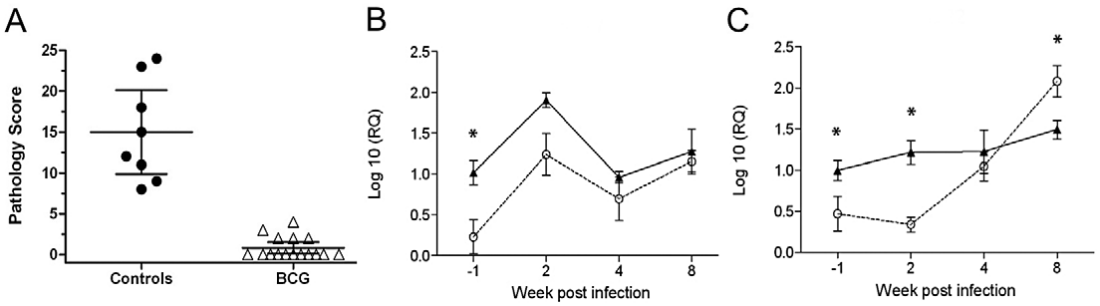
Gene expression in PPDB-stimulated PBMC from BCG vaccinated and control cattle. A. Protective efficacy after *M. bovis* challenge determined by pathology scoring. Results are expressed as total pathology scores [5]. Filled circles, unvaccinated control calves; open triangles, BCG vaccinated calves. (B, C). Transcription of the genes expressing IFN-γ (B) and IL-22 (C) following *in vitro* stimulation. PBMC were collected from BCG vaccinated (filled symbols) and controls (open symbols) before challenge (week -1) and after challenge with *M. bovis* at weeks 2, 4 and 8, and stimulated with PPD-B for 24 hours. cDNA was prepared and gene expression determined by RT-qPCR. Data are expressed as log10 relative expression levels compared to non-stimulated cells. Statistical analysis: 2-way ANOVA with Bonferroni post test, * P<0.05.

The outcome of this experiment in respect to protective efficacy is shown in Figure 6A. All BCG vaccinated animals in this experiment were protected and we therefore compared the transcription of *il-22* from control animals with those obtained from BCG vaccinated animals using RT-qPCR, transcription of *ifn-γ* was used as control. PBMC were prepared and stimulated with PPD-B at 4 time points: Immediately prior to *M. bovis* infection (week –1) and 2, 4, and 8 weeks post-challenge. Confirming the results obtained by RNA-seq, *il-22* was up-regulated significantly after vaccination and prior to challenge (Figure 6C, week –1, P<0.05) as was *ifn-γ*. Interestingly, IL-22 transcription increased post-infection in the unvaccinated calves, as we have previously shown [13]; a pattern that was mirrored in the BCG vaccinated animals. Consequently, no differences between *il-22* transcription between the two groups were observed post-*M. bovis* infection (Figure 6C). As it is possible that different cell populations are producing IL-22 at the different time points pre and post-challenge, we are currently phenotyping responder populations. Data so far indicate, that at least in infected, non-vaccinated cattle, IL-22 is transcribed exclusively in CD4^+^ T cells [13]. As expected, transcription of *ifn-γ* was also significantly increased after vaccination (week –1, Figure 6B) as well as immediately after infection (week 2) (P<0.05, Figure 6B). In contrast *ifn-γ* transcription in unvaccinated animals developed slowly post-infection at week 4, and was significantly higher in unvaccinated animals than in BCG vaccinated at week 8 post-vaccination (P<0.05, Figure 6B). In summary, the results from this independent experiment confirmed the role of il-22 transcription in predicting protective immunity.

## Discussion

The design and development of a new bTB vaccine, would be greatly facilitated by the definition of predictors and correlates of protection, which could potentially be identified by studying the vaccine-induced host responses after vaccination and both before and post *M. bovis* challenge. In particular, their definition would allow rational vaccine design and testing thereby reducing the costs of large animal experiments, as well as speeding up the testing process, by selecting the most promising candidate vaccines to be challenged with *M. bovis* in cattle BSL3 facilities. Such an evidence-based gating strategy would allow a faster and more informed rational approach to TB vaccine development. However, the classic reductionist scientific approach in hypothesis validation using conventional immunological methods has severe limitations when it comes to scan, identify and define thousands of potential biomarkers in parallel. Thus, systems biology, based in the integration of data generated by –*omics* studies, has emerged as a useful approach in order to identify gene signatures that can predict and/or correlate with protection following vaccination [15].

Although the immune response against TB is thought to be based primarily on the key TH1 cytokine IFN-γ, its induction following vaccination does not necessarily demonstrate vaccine success. However, absence of its expression after vaccination can indicate the failure of the vaccine tested to protect against bTB [8], [9]. Thus, the expression of IFN-γ following vaccination alone is insufficient as a predictor of protection. In an earlier study, the transcription of IL-17A predicted protection following vaccination with BCG and correlated with protection after challenge with *M. bovis* [12]. Although elevated IL-17A transcription was also found in the present study in PPD-B stimulated PBMC from vaccinated/protected compared to vaccinated/un-protected calves, the differences did not quite reach statistical significance (data not shown).

Another cytokine that is associated with a TH17 responses is IL-22 [16] (although IL-22 can also be induced in an TH17-independent manner by TH22, for example [17]), which is involved in barrier surface protection and healing in skin, intestine and lung [16]. In the present study, *il-22* was the gene most up-regulated in vaccinated/protected animals and thus constitutes a surrogate of protection. To our knowledge, this is the first report describing a role for IL-22 in vaccination against bTB. Previously, it had been shown that IL-22 produced by NK cells in humans and CD4^+^ T cells in macaques could limit *M. tuberculosis* growth in macrophages by increasing phagolysosomal fusion [18], [19]. However, IL-22 can play a dual role in tissue homeostasis depending on the cytokine microenvironment where it is induced. For example, Sonnenberg *et al.* showed that excessive pulmonary destruction after intratracheal administration of bleomycin induced IL-22, whilst in IL-17 ^−/−^ mice, IL-22 showed tissue protective properties [17]. Further, a murine model of allergic asthma using OVA immunization in IL-22-deficient mice showed reduced lung inflammation during the sensitization phase but increased lung inflammation during the antigen challenge (inhibited by exogenous IL-22) [20]. Thus, these opposing biological effects might explain in part why IL-22 in our previous study was associated with pathology in unvaccinated naturally infected cattle [13], whilst in this study our data suggest that BCG vaccination induced an adequate and balanced inflammatory and TH1/TH17 (or TH22) cytokine response, with the expression of IL-22 in BCG vaccinated cattle maintained at relatively stable levels after infection compared with the increased observed in unvaccinated controls at week 4 and 8 p.i., as demonstrated in our validation experiment where we measured *il-22* transcription also after *M. bovis* challenge.

Besides the genes encoding for IL-22 and IFN-γ a number of other genes were strongly and significantly regulated exclusively in vaccinated/protected calves. For example *mt3*, encoding the zinc metallothionein MT-3 was the second most up-regulated gene in this category after *il-22*. This protein is a zinc-binding protein whose role in lysosomal function and autophagy of for example neurons and astrocytes has been described [21]. Autophagy has been linked with innate and adaptive immune responses against intracellular pathogens including *M. tuberculosis*, and *mt3* up-regulation could reflect IFN-γ induced macrophage activation. Interestingly, the transcription of the TH2-associated cytokine *il-13* is also up-regulated, which is a cytokine that inhibits autophagy alongside IL-4 [22]. The gene encoding for the chemokine CCL3 (MIP1α) was also found to be strongly up-regulated in PBMC from protected calves. CCL3 is produced by innate cells as well as CD4+ T cells during human TB (e.g. [23], [24]). Its role after vaccination may be to recruit antigen-specific T cells into the airway lumen following infection with *M. tuberculosis* or *M. bovis* [25]. *Ccl3* in PPD-B stimulated PBMC may be a reflection of the strong IFN-γ responses induced.

In summary, although the development of TH1 responses following vaccination is fundamental for TB vaccine success as has been confirmed in this study by the significant modulation of IFN-γ expression as well as IFN-γ induced genes, up-regulation of the gene encoding *il-22* as the dominant gene predicting vaccine success has been the major finding of this study. Thus, induction of TH17/22 subset responses alongside TH1 responses appears to be important for the protective anti-tuberculosis response. However, this biosignature needs to be prospectively validated in future experiments to assess its full potentials for predicting protection.

## Materials and Methods

### Ethics

This study and all procedures were approved by the Animal Health and Veterinary Laboratories Agency (AHVLA) Animal Use Ethics Committee (UK Home Office PCD 70/6905) and performed under appropriate personal and project licences within the conditions of the Animals (Scientific Procedures) Act 1986. All animals were housed in appropriate biological containment facilities at the AHVLA.

### Animals

Holstein-Friesian calves used in this study were recruited from tuberculosis-free GB farms either neonatally or when around 6 months old. All animals were housed in appropriate biosafety levels containment facilities and allowed access to food and water *ad libitum*.

### Vaccines

*Mycobacterium bovis*-Bacillus Calmette-Guérin (BCG) Danish strain 1331 SSI (Serum Staten Institute, Copenhagen, Denmark) was used for immunisations within 1 hour of vaccine reconstitution as per manufacturer's instructions. The vaccine was prepared fresh from lyophilised stock on the day of vaccination according to the manufacture's instructions and administrated subcutaneously in a 0.5 ml volume. Each BCG vaccinated calf received the equivalent of 5 human doses which on culture was determined to be approximately 10^6^ CFU. A recombinant human type 5 adenovirus expressing the mycobacterial antigen Ag85A (Ad85) was used at 2×10^9^ infectious units/dose and applied via the intradermal route [12].

### Challenge and *post mortem* procedures

Calves were challenged with 2000 CFU *M. bovis* strain AF2122/97 [26] by the endobronchial route. The infection inoculum was grown to mid log phase in Middlebrook 7H9 broth supplemented with 4.16 g/L pyruvic acid, 10% (v/v) oleic acid, albumin, dextrose, and catalase (OADC) and 0.05% (v/v) Tween 80, and subsequently stored at −80°C until being used in the experimental infection experiments. At 9 or 12 weeks post-infection, cattle were euthanized and underwent detailed *post mortem* examination; the severity of the pathological changes after infection were scored using the system described before [5].

### Vaccination, blood collection and challenge schedules

*Experiment 1:* 6 months old calves were vaccinated with BCG as described above; 8 weeks later, a subset of these animals was boosted with Ad85. All animals were challenged 6 weeks later with *M. bovis* AF2122/97 as described above and their disease status determined 12 weeks later by post mortem examination as described above. Blood was collected for analysis 14 weeks post-BCG vaccination (and 6 weeks post-Ad85A boost) immediately prior to *M. bovis* infection.

Experiment 2: Neonatal calves were vaccinated with BCG when under 6 weeks old (as described above). 7 months later animals were challenged with *M. bovis* AF2122/97 and their disease status determined by post-mortem examination as described above. Blood was collected 1 week prior to *M. bovis* infection and 2, 4, and 8 weeks post-challenge.

### Bovine Peripheral Blood Mononuclear Cells (PBMC)

PBMC were isolated from heparinized blood collected before *M. bovis* challenge by Histopaque-1077 (Sigma-Aldrich) gradient centrifugation and resuspended at 2×10^6^/ml in tissue culture medium (RPMI 1640 [Sigma] supplemented with 10% fetal calf serum [Sigma], nonessential amino acids [Sigma], 100 U/ml penicillin and 100 µg/ml streptomycin sulphate [Gibco]) and incubated overnight with bovine tuberculin (PPD-B, 10 µg/ml, Prionics, Schlieren, Switzerland) in 24-well tissue culture plates in 1 ml aliquots (Life Technologies). On the following day, plates were centrifuged (300×*g*, 5 min at room temperature) and the supernatants removed. For experiment 1, samples were responded in 0.3 ml GTC (4 M guanidine thiocyanate, 25 mM tri-sodium citrate pH 7, 0.1 M 2-mercaptoethanol, 0.5% Tween 80, 0.5% sodium N-lauryl sarcosine). In experiment 2, 1 ml of Trizol (Invitrogen) was added. Cell lysates were stored at −80°C.

### DNA library preparation and sequencing

DNA library preparation and sequencing was done according to manufacturer's instruction using mRNA-Seq-8 Sample Preparation Kit (Illumina, San Diego, CA). Briefly poly-A containing mRNA was isolated and purified from total RNA using poly-T oligo-attached magnetic beads. The purified m-RNA was fragmented using divalent cations at 94°C for 5 minutes. After the first strand cDNA synthesis using reverse transcriptase and random hexamer primers, the second strand cDNA synthesis was performed using DNA polymerase generating double-stranded cDNA. RNA was digested with RNaseH, then cDNA was purified using QIAquick PCR Purification Kit (Qiagen). The purified cDNA fragments were end repaired to convert the 5′ and 3′ overhangs into blunt phosphorylated ends using T4 DNA polymerase and Klenow DNA polymerase before adding a single “A” base to the 3′ end of the blunt phosphorylated cDNA fragments using 3′-to-5′ exo-nuclease. Adaptors were ligated to the ends of the cDNA fragments. Size selection was done using 2% agarose gel. Approximately 200 bp cDNA was excised and gel purified then enriched by PCR amplification for 15 cycles. Each library was quantified using Agilent DNA 1000 kit (Agilent) on Agilent 2100 Bioanalyzer. These libraries were denaturated using NaOH and diluted to a final concentration of 6 pM. 100 µl of these diluted libraries were used on Cluster Station using Clustering Generation Kit v4 (Illumina). Sequencing was done on Genome Analyzer IIx by singe end sequencing using a 36 Cycle Sequencing Kit v4 (Illumina). Image analysis and base calling was done using Genome Analyzer Pipeline software v1.5.0 (Illumina) to generate raw fastq files.

The short sequence reads were assembled using the CLC Genomics Workbench 4.7.2 RNA-Seq Analysis application using the *Bos taurus* genome (Baylor4/bosTau4) [27] as reference with a maximum of two mismatches and ungapped alignment. The normalization of the assembled data was calculated with the reads per kilobase of exon model per million reads (RPKM) [28] and statistical difference in expression levels was calculated using Baggerley test [29] using CLC Genomics Workbench. Only genes that were significant regulated (p<0.05) more than ±2 fold change were selected for pathway analysis using KEGG mapper [30], and further analysis using the Database for Annotation, Visualization and Integrated Discovery (DAVID) [31], [32]. The raw data of the experiment was submitted to the NCBI Sequence Read Archive (www.ncbi.nlm.nih.gov/sra/) with accession number SRA054204.

### RNA extraction and Quantitative Real-time PCR

Total RNA was extracted from PBMC using TRIzol (Invitrogen) according to the protocol recommended by the manufacturer. Turbo DNA-free (Ambion, Foster City, CA, USA) was used to remove genomic DNA contamination. The purity and concentration of RNA were evaluated by NanoDrop 1000 (Thermo Scientific, Ottawa, Canada). cDNA from PBMCs was synthesized from total RNA samples using random primers and reverse transcription with Transcriptor High Fidelity reverse transcriptase enzyme following the manufacturers protocol (Roche, Basel, Switzerland).

#### Quantitative Real-time PCR

Transcripts were quantified by qPCR with Fast SYBR Green master mix (Applied Biosystems California, USA) following the manufactures instructions. qPCR analysis was performed in triplicates using an ABI 7500 Fast Real Time PCR System (Applied Biosystems California, USA). The fold increase was calculated by comparison with the expression of the endogenous controls genes SDHA, YWHAZ (bovine) [33] and G3PDH using the 2^−ΔΔct^ calculation [34], [35].

## Supporting Information

Table S1 **List of genes significantly (p<0.05) modulated in vaccinated and protected calves compared to naïve and vaccinated/unprotected animals.** PBMC were isolated from prior to *M. bovis* infection and stimulated with bovine PPD-B. In ‘weighted proportions fold change’ column strength of red colour indicate degree of up-regulated, of green, down-regulated genes.
(XLS)
